# Nutritional therapy and infectious diseases: a two-edged sword

**DOI:** 10.1186/1475-2891-5-21

**Published:** 2006-09-04

**Authors:** Haig Donabedian

**Affiliations:** 1 Professor of Medicine, College of Medicine, University of Toledo, 3120 Glendale Avenue, Toledo, OH 43614, USA

## Abstract

The benefits and risks of nutritional therapies in the prevention and management of infectious diseases in the developed world are reviewed. There is strong evidence that early enteral feeding of patients prevents infections in a variety of traumatic and surgical illnesses. There is, however, little support for similar early feeding in medical illnesses. Parenteral nutrition increases the risk of infection when compared to enteral feeding or delayed nutrition. The use of gastric feedings appears to be as safe and effective as small bowel feedings. Dietary supplementation with glutamine appears to lower the risk of post-surgical infections and the ingestion of cranberry products has value in preventing urinary tract infections in women.

## Background

One of the earliest responses to infection is cytokine-mediated anorexia. Interleukins 1, 6, 8 as well as tumor necrosis factor and interferon alpha are released by host defense mechanisms. These cytokines reduce nutrient intake through effects on the central nervous system [1]. They also cause the sequestration of critical nutrients such as iron, copper and zinc in order to allow the host to gain an advantage over invading organisms [2]. Is it a benefit to bypass the action of the body's host defense mechanisms by feeding patients who do not or cannot ingest their normal diet?

The developed world is rife with calories and nutrients. Is there a benefit from nutritional supplements in preventing infections in societies where the availability of nutrients is not limited?

Physicians are often asked about the role of various nutritional interventions in preventing or treating infectious diseases. What is known about the value of these interventions?

This review will not deal with nutritional interventions in populations which often suffer dietary deficiencies. As such, it will include only studies done in economically developed nations. Finally, it will review the use of cranberry products and yogurt as these are available in any grocery store, but not the myriad of botanical products sold for their medicinal value.

In order to clarify what is known about the risks and benefits of nutrition in the developed world, I have reviewed the English language publications available on MEDLINE (1966–2006) and in the Cochrane Library which deal with nutrition and infection. All articles including the words "nutrition", "vitamin(s)", "cranberry" or "yogurt" and the words "infection" or "infectious" in their abstracts were reviewed. Articles were included if they were structured to compare differing nutritional interventions.

### Prevention of infection in the general population and the institutionalized

Is nutritional therapy of value in preventing infectious diseases in the developed world? There is a large body of evidence that supports the thesis that insufficient intake of dietary protein adversely affects the immune system and predisposes the malnourished to infectious diseases [3-5]. This inadequate protein intake is usually coupled with reduced intake of calories and is referred to as protein-calorie malnutrition (PCM).

#### Protein

Since there are no prospective studies in which protein intake is manipulated in order to assess infection risk in non-hospitalized people, indirect studies must be evaluated. The use of serum albumin as an indicator of protein intake is fraught with problems as chronic illnesses in themselves may prevent adequate protein intake and may inhibit the synthesis of albumin [6].

Preoperative albumin levels have been shown to correlate with the postoperative risk for pneumonia, urinary tract infections and wound infections in a large Veterans Administration study [7]. This study did not deal directly with nutrition, however. A more relevant study looked at 1,023 acute trauma patients admitted to a Baltimore hospital. Since these patients were acutely ill due to a non-medical condition, their albumin levels were more likely to reflect previous nutritional status rather than previous medical illness. There was a 48% incidence of infection in those admitted with a serum albumin of less than 2.6 gms/dL and a 28% incidence in those with an albumin of 2.6 gms/dL or above (p < 0.001). The infections were predominately respiratory and urinary in nature [8].

This evidence indirectly supports adequate protein intake as a factor in preventing infections after trauma.

#### Multivitamins and zinc

Studies have shown that most elderly patients fail to ingest the recommended daily allowance (RDA) of zinc. Supplementation with zinc could improve their immune responses to infection and thus prevent illness. Zinc supplementation for people aged 60–89 (defined as elderly) alone increased their in vitro lymphocytes' response to mitogens [9]. Their response to skin test antigens however, was not increased. When 15 mg of zinc was added to a multivitamin preparation and compared to a lactose placebo given to people aged 59–85 living in northern New Jersey, there was a significant increase in skin test responses to a panel of seven intradermal antigens [10]. The incidence of infectious diseases was not studied.

There is a considerable literature concerning the use of zinc as a therapy for the common cold. Marshall has reviewed 8 such studies and concludes that there is no convincing evidence as to zinc's efficacy in reducing the severity or duration of cold symptoms [11].

There is evidence that zinc supplementation significantly reduces the incidence of infections in people with sickle cell disease. Twenty-one of 32 sickle cell disease patients in the Detroit area were found to have zinc deficiency as determined by lymphocyte or granulocyte zinc levels. Zinc supplementation in those deficient reduced their documented infection rate by as much as 80% [12]. Their hospitalization rate, however, was unaffected.

Multivitamin and mineral supplementation has not been shown to affect illness or absenteeism rates in 158 adults (age >45 years old) living in North Carolina unless they had type II diabetes [13]. Diabetics taking a placebo reported a 93% incidence of infection (as opposed to an incidence in non-diabetics of 60%). Strangely, diabetics taking supplementation showed only a 17% incidence of infection-much lower than non-diabetics taking placebo. The infections did not result in hospitalizations. Elderly people (>60 years old) living in central France had no decrease in infection incidence when receiving a multivitamin supplementation as compared to a placebo [14]. A prospective study relating the intake of carotenoids, vitamins C and E and B-vitamins with the incidence of community acquired pneumonia in over 50,000 U. S. male health professionals aged 40–75 years old failed to show any correlation between pneumonia risk and vitamin intake [15]. This study looked at both food and supplement sources of vitamins. "Natural" vitamins therefore seemed to be no better than "pharmaceutical" vitamins in preventing pneumonia in this well-educated population of American men. Elderly nursing home residents in the Boston area were given multivitamins with either 4 IU of vitamin E (50% of the RDA) or 200 IU of vitamin E. High intake of vitamin E had no effect on the incidence or duration of lower respiratory tract infection [16]. There appeared to be a small, but significant effect on the incidence of upper respiratory tract infections (including otitis media), but not on their duration (risk ratio = 0.84 for incidence, but 1.53 for duration).

There are two studies which support the use of vitamin or micronutrient supplementation to prevent community-acquired infections. Chandra [17] showed that trace elements and multivitamins reduce the number of days with any sort of infection. The study enrolled 96 elderly Newfoundland residents and is apparently the only other study to show such an effect in those not institutionalized.

A French study gave a placebo, zinc and selenium supplements, or multivitamins with zinc and selenium to institutionalized people over the age of 65 [18]. The sample size was a total of 81 and over a two year follow-up period, they found that the number of pneumonias and UTIs decreased by about 50% in those who received the zinc and selenium with or without multivitamins. The multivitamins had no statistically significant effect alone.

If there is little or no effect of micronutrient and multivitamin supplementation on infection rates on apparently uninfected members of the community, how about those chronically infected with viruses? A small study from the era prior to the availability of effective anti-HIV therapy studied nutrient and vitamin intake in 56 HIV infected New Yorkers. Vitamin intake varied from 2% to 50,000% of the RDA. No correlation could be found between nutrient intake and CD4 lymphocyte count or absolute lymphocyte count [19].

A more intriguing study of HIV-infected people in Baltimore correlated their micronutrient intake as estimated by data from a questionnaire and the subsequent progression of HIV disease [20]. This study did not take any changing dietary habits into account. The study correlated any intake of zinc supplements with decreased survival. High intake of vitamins B_1_, B_2 _and B_6 _were correlated with increased survival. Studies of this nature always raise the question of whether the increased intake reflected a healthier life style or produced a healthier life. A review of fifteen studies utilizing vitamin and micronutrient supplementation in HIV-infected people concluded that such supplementation effected no reduction in mortality or morbidity in HIV-infected adults. HIV-infected children in under-developed countries could benefit from vitamin A supplementation, however [21].

Hepatitis C has a prevalence between 1% and 2% in the U. S. population. Could nutritional therapy affect the progression to cirrhosis or hepatoma? The only vitamin supplementation studies done with hepatitis C involve the use of vitamin E as an anti-oxidant to limit hepatic fibrosis [22]. Two studies which show decreased transaminase levels when patients with hepatitis C are given supplemental vitamin E [23,24] although there is no data on vitamin E's effect on the viral load.

#### Vitamin C

The RDA for vitamin C is between 45 and 60 mg per day. The writings of Linus Pauling have popularized the use of vitamin C as a prophylactic and therapeutic agent for the common cold [25]. The nutritional value of a vitamin ingested at more than 10 times its RDA begs the question of whether the vitamin is nutritional or pharmacologic. A comprehensive meta-analysis has been published [26] which finds that huge doses of vitamin C have a minimal effect on reducing cold symptoms. No effect on cold prevention could be found unless groups undergoing hypothermic stress were studied.

There is a published study which supplemented British patients over the age of 65 with a placebo or 200 mg of vitamin C if they were admitted to hospital with bronchitis or pneumonia [27]. The patients were followed for 4 weeks. If patients who died were excluded from the analysis, the study showed that vitamin C administration lessened the symptom score of surviving patients. Only one of the six deaths in the study occurred in a vitamin C recipient, but the sample size was too small (n = 57) to show significance.

#### Cranberry juice

Many people believe that drinking cranberry juice will treat or prevent urinary tract infections. There is evidence that cranberry juice contains anti-adhesive molecules which could interfere with bacterial virulence mechanisms [28]. Cranberry juice (300 ml/day) or a colored drink containing no cranberry products were provided to female residents of a long-term care facility in Boston [29]. Monthly urine samples were analyzed. At one month, the percentage of urine samples with more than 100,000 CFU/ml of urine was identical whether or not cranberry juice was ingested. After one month, there was a consistent decrease in high-grade bacteriuria in the cranberry juice drinkers. The calculated relative risk for bacteriuria with pyuria for cranberry drinkers was 0.42 (p = 0.0004). Antibiotic use for UTI was almost halved in those drinking cranberry juice. In this study, the distinction between UTI prevention and treatment is unclear, but its ability to obviate the use of antibiotics is significant.

A Canadian study randomized 150 sexually active women who had ≥ 2 UTIs in the previous year into three groups: 1) 250 ml of diluted pineapple juice (colored red) thrice daily and a placebo tablet twice daily; 2) A concentrated cranberry extract tablet twice daily and placebo juice; 3) 250 ml of cranberry juice thrice daily and a placebo tablet [30]. The study continued for a year. Symptomatic infections were treated with antibiotics for 3 days and the prophylaxis restarted. The placebo group showed 32% of entrants with at least one UTI during the year of study. The tablet group had 18% with at least one infection and the cranberry juice group had 20%. The difference between the tablet or juice groups and placebo was significant (p < 0.05). The mean number of UTIs per year was approximately halved by the use of a cranberry product.

The daily ingestion of cranberry juice concentrate or a placebo did not affect bacteriuria rates in children requiring intermittent catheterization [31].

There is sufficient reason based on these two positive studies and others reviewed by Raz et al [29] to recommend a cranberry product to women with recurrent UTIs.

#### Yogurt

Yogurt is cow's milk usually fermented using two synergistic bacterial species *Lactobacillus delbrueckii *subspecies *bulgaricus *and *Streptococcus thermophilus*. The amount of fat and even the bacteria used to ferment the milk may vary from study to study. The presence or absence of living bacteria must be kept in mind when evaluating studies which employ yogurt. There are few randomized studies in which yogurt (as opposed to lactobacilli or yeast in pure form) is administered in the developed world. Beniwal *et al *gave 109 American patients 227 grams of vanilla flavored yogurt twice daily for 8 days if they were receiving intravenous or oral antibiotics [32]. The yogurt contained *L. acidophilus *as well as *L. bulgaricus *and *S. thermophilus*. The control group of 97 patients received no yogurt. The mean number of days of yogurt intake was 6.6 days. Diarrhea as defined by 3 or more loose bowel movements per day was reported in 13% of yogurt ingesters and 23% of those not eating yogurt. The duration of diarrhea was not significantly decreased. Israeli soldiers eating yogurt with living *L. casei *did not have fewer diarrheal episodes than those soldiers eating yogurt containing no living bacteria [33]. The study had approximately 250 soldiers in each arm and the mean incidence of diarrhea was 14%. If there were an undetected effect, it would have had to be a small one.

While not a nutritional therapy per se lactobacilli are used by those who do not tolerate yogurt or find it unfeasible. *Lactobacillus casei *strain GG was administered in capsules (not yogurt) to American children receiving concurrent antibiotics. The living bacteria reduced the percentage of children with diarrhea (defined as more than one liquid stool/day) from 26 to 8% when compared to an inulin placebo [34]. A Finnish study using *Lactobacillus *strain GG found that recovery from diarrhea was one day quicker on average in those children that received living bacteria in capsular form as opposed to children who received a placebo [35]. The same *Lactobacillus casei *strain reduced the incidence of antibiotic-associated diarrhea in Finnish children from 16% to 5% [36].

When the effect of the same GG strain was used to prevent antibiotic associated diarrhea in American adults, the results were disappointing. A study analyzed 268 hospitalized patients and found almost identical diarrhea rates and frequencies in those receiving the live bacteria and in those receiving a placebo [37].

The "non pathogenic" yeast *Saccharomyces boulardii *has been shown to reduce antibiotic-related diarrhea in hospitalized Americans when administered in capsule form [38]. Enthusiasm for this therapy has waned due to reports such as that of 7 French patients who developed fungemia after ingesting this yeast in the setting of an intensive care unit [39].

The ability of yogurt or lactobacilli to prevent diarrhea is still in question. Should yogurt contain living bacteria? Do the species of bacteria matter? Can yogurt be made with skim milk? Does the flavoring matter? How much yogurt should be ingested? These questions probably will never be answered. At present, yogurt cannot be recommended as a proven method of diarrhea prevention.

Table 1 summarizes the effects of various nutritional interventions on infections in the general population and in institutionalized people.

**Table 1:** Summary of randomized, controlled trials of the impact of nutritional interventions on infections in the non-hospitalized population

**Intervention**	**Effect**	**Reference**
Zinc supplementation	No effect on common colds	11
Zinc supplementation	reduced infection incidence in sickle cell patients	12
Zinc and selenium supplementation	Reduced infection incidence	18
Vitamin and mineral supplementation	Reduced infection incidence in type II diabetics only	13
Vitamin and mineral supplementation	Reduced infection incidence	17
Multivitamin supplements	No effect on infection incidence	14
Multivitamin supplements	No effect on pneumonia incidence	15
Vitamin E supplements	Reduced URI rate, no effect on lower respiratory tract rate	16
Vitamin C therapy or supplements	No effect on common cold unless used in hypothermic conditions	26
Cranberry juice	Reduced incidence of bacteriuria	29, 30
Yogurt	Reduced diarrhea incidence in adults taking antibiotics	32
Yogurt	No effect on diarrhea incidence	33
Lactobacilli	Reduced incidence of diarrhea in children	34–36
Lactobacilli	No effect on diarrhea incidence in adults	37

### Pre-operative nutrition

If the value of nutritional supplementation in the general citizenry of developed countries is unproven, how about for those about to undergo surgery?

A pre-operative cohort of 192 malnourished American veterans were randomized to receive total parenteral nutrition (TPN) and 203 control malnourished patients received conventional nutrition [40]. Malnutrition was defined by serum albumin or prealbumin levels, by a body weight ≤ 95% of ideal or a score ≤ 100 on the Nutrition Risk Index. The Nutrition Risk Index uses body weight and serum albumin to calculate a malnutrition score. The TPN patients received therapy for at least seven preoperative days whereas the conventional patients went to surgery within 3 days. The TPN patients received TPN postoperatively for at least 72 hours. Conventional patients received no parenteral or enteral post operative feedings for 72 hours. After that time, they could receive whatever form of nutrition thought best. Overall infectious risk in a 30-day post operative period was 14.1% in the TPN group and 6.4% in the conventional group (p = 0.01). The bacteremia/fungemia rates for TPN patients were 4.2% vs 2.5% and the rate of pneumonias was almost double that of the conventional group when patients were stratified by degree of malnutrition. The most severely malnourished TPN patients had a 12.9% infectious complication rate as opposed to a 10.5% rate in the severely malnourished conventional patients. The preoperative TPN course did not therefore ameliorate the infectious risk-if anything it exacerbated it.

### Early vs. late nutrition

Patients who have undergone trauma or surgery need to be fed eventually. Would early feeding prevent or increase the rate of infections? There are several studies that examine various subsets of these patients.

Nasojejunal tubes were placed in 17 Arizona head trauma patients and feeding started within 36 hours of admission [41]. Feeding of the 15 control patients was by the nasogastric route and never before 72 hours. The patients were studied only for 7 days after admission. In this short time period, there were 14 infections in the late feeding group and only 3 in the early feeding group. The predominant infection was "bronchitis" – a diagnosis whose criteria were not defined. Since this study was not blinded, observer bias would be hard to eliminate. A similar study was done in Tennessee using 30 patients fed nasogastrically before 72 hours or after gastric ileus was resolved [42]. There was no difference in the incidence of pneumonia or infections in general. A small study enrolling cervical trauma patients also failed to show an advantage with early enteral feeding [43].

Sixty-three abdominal trauma patients requiring celiotomy were entered into a study in which half the patients received intravenous glucose for the first five post-operative days and the other half had an immediate percutaneous jejunal feeding tube placed through which was administered an elemental diet [44]. Patients fed immediately had a "sepsis" rate of 9% while those not fed for five days had a rate of 29% in the first seven days (p < 0.05). Unfortunately, "sepsis" was not further defined. A meta-analysis of early feeding in patients undergoing gastrointestinal surgery included 11 studies and 837 patients [45]. The patients were fed either orally or by jejunal feeding. Early was defined as within 24 hours of surgery. The relative risk of all infections was 0.72 in favor of early feeding. The risk of pneumonia was between 0.7 and 0.8 and the risk of intra-abdominal abscess was between 0.8 and 0.9. The rationale behind early enteral feeding in gastrointestinal surgery is based on the putative increase in intestinal epithelial health and decreased translocation of intestinal flora into the systemic circulation. Numerous studies seem to confirm early enteral feedings' value in abdominal surgery, whether or not the rationale is valid. The benefit of early feeding has not been found in acutely burned children who were fed within 24 hours of the burn as opposed to after 48 hours [46], but the mean difference of 33 hours in delaying nutrition cannot be said to be large.

A review of 15 studies incorporating abdominal surgery, trauma, head injury and burns came to the conclusion that early (within 36 hours) enteral feeding yielded a significantly decreased rate of infection (19% vs 41% p = 0.049). There was considerable heterogeneity amongst the studies, with abdominal surgery studies having the most convincing risk reduction [47].

The benefit of early feeding in trauma and surgery patients does not appear to generalize to early enteral feeding in those with severe medical illnesses.

The FOOD study looked at the benefit of early tube feeding in dysphagic stroke patients from around the world [48]. Four hundred and thirty patients were randomized to avoid any enteral tube feedings for 7 days as opposed to 429 allocated to nasogastric or percutaneous endoscopic gastrostomy (PEG) feeding within 3 days of enrollment. Early feeding had no effect on the rates of pneumonia or urinary tract infections. Nor was there an effect on survival by delaying feeding for seven days.

All patients ventilated in a St. Louis ICU received an orogastric tube. Early-fed patients (n = 75) received their nutritional requirements from day 1, while the late fed (n = 75) patients received 20% of their requirements for 4 days and then their feedings were increased to their calculated optimal level [49]. The early feeding group had no fewer bacteremias or UTIs. The early feeding group had 65% more antibiotic days than the late feeding group (p < 0.001) and their length of stay in the ICU was almost twice as long. Ventilator-associated pneumonias occurred in 37% of the early feeding group as opposed to 23% of the late feeding group (p = 0.02). The size of this study and its' quality argues against an infectious benefit by early feeding of medical patients requiring ventilation.

### The route of nutrition delivery

Is there significant benefit or risk associated with the route chosen for delivery of nutrition? Would total parenteral nutrition (TPN) increase the bacteremia rate, but decrease the incidence of aspiration pneumonia, for instance? Could jejunal feeding reduce the likelihood of esophageal reflux and thus lower the pneumonia rate when compared to gastric feeding?

#### Gastric vs non-gastric enteral feeding

Gastrostomy feeding of 10 neurologically dysphagic patients was compared to 20 fed by nasogastric tube in Scotland [50]. The nasogastric tube was a "fine bore" 20 French tube. After 28 days, the gastrostomy patient group suffered two aspiration pneumonias and one wound infection versus no pneumonias for the nasogastric group. Another small study compared ventilated Canadian trauma patients receiving gastric versus duodenal feedings via nasal or oral tubes. Forty three gastrically fed patients had 18 clinically diagnosed pneumonias versus 10 pneumonias in the 37 patients receiving duodenal feedings. No difference was statistically discernible with the sample size used. Nasogastric and nasoduodenal feeding of ventilated patients was compared in 44 California medical ICU patients [51]. The pneumonia, bacteremia and mortality rates were indistinguishable.

Jejunal feedings were compared to gastric feedings in 38 Boston medical and surgical ICU patients. The jejunal tubes were placed perorally. About half of each group was ventilated. Two gastrically fed patients developed pneumonia versus none of the jejunally fed [52]. Such a small study is not convincing.

Two meta-analyses have been published which analyzed the differences between gastric and non-gastric enteral feeding. One [53] found a relative risk of 0.76 of developing a pneumonia for small bowel (duodenal or jejunal) feedings. The other [54] found no proven benefit in preventing pneumonia by employing small bowel feedings. The consensus statement of a North American summit on aspiration in critically ill patients likewise concluded that good evidence does not exist for small bowel feedings unless proven aspiration occurs and then jejunal feedings were recommended [55]. This consensus group did recommend continuous enteral feeding rather than bolus feeding apparently based on the study carried out on 60 Nebraskan patients receiving nasogastric feedings [56]. This study only followed elderly patients for seven days. All patients were assessed clinically for aspiration and continuously fed patients had one-half the aspiration rate of intermittently fed patients. This difference was not statistically significant.

#### TPN vs enteral nutrition

There are many small trials in various sub-groups of patients (i.e.: lymphoma, radiotherapy) comparing enteral and total parenteral nutrition. This review will discuss only meta-analyses of various subgroups for the sake of brevity as the findings are remarkably homogenous. A large meta-analysis of 7 studies comparing TPN with standard care and 20 studies comparing tube feeding to TPN was done by Braunschweig et al [57]. A total of 1,828 patients were included in these randomized prospective studies. Standard care patients had a relative risk of infection of 0.77 when compared to TPN and enterally fed patients had a relative infection risk of 0.66. If catheter infections were excluded from the meta-analysis, the increased risk of infection associated with TPN persisted.

A much smaller meta-analysis using mostly unpublished studies was done on 230 surgical and trauma patients [58]. The overall risk of infection complications was 2.5 times higher (p = 0.0001) in those randomized to TPN as opposed to enteral feedings early in the course of hospitalization. Rather than increasing the risk of aspiration and pneumonia, enteral feedings reduced that overall risk of pneumonia to 40% of that seen in TPN patients.

Since pancreatitis causes severe inflammation which may lead to transluminal spread of bacteria, it would be reasonable to suppose that TPN would decrease the infectious risks in patients with pancreatitis by decreasing pancreatic activity. The combined risk for infection for pancreatitis patients in two trials (n = 70) of enteral feeding was 0.61 when compared to the TPN route [59].

Chemotherapy patients may have long periods of anorexia and poor nutrition. Would TPN mitigate this malnutrition and allow a more vigorous defense against infection? Many small studies examining this question were published in the 1980's and 1990's. None showed an infection benefit with TPN. A meta-analysis of 15 studies comparing TPN with routine care was published in 1990 [60]. Three month survival and chemotherapy response were not improved by the use of TPN. The risk of infection was 3.2 times higher in those receiving TPN (p < 0.005) even if catheter-related infections were excluded from the analysis. If catheter infections were included, the risk of infection was 4 times greater in those receiving TPN. A recent Brazilian study [61] found that a central venous catheter through which TPN was given had a 3.3 times greater risk of becoming infected than a similar catheter not used for TPN.

Is the flood of intravenous nutrients responsible for the increased rate of infections seen with TPN or is it the lack of enteral feeding and the presence of a central venous catheter? Some insight into the answer is gained by the result of studies comparing TPN with intravenous feedings of other types.

A study of 248 Swedish post-operative patients were randomized to TPN or glucose and electrolyte solutions until they could eat and drink "freely" without the use of any intravenous support [62]. They found that those supported with glucose and electrolytes had a 5% mortality as opposed to 2% with TPN, but this difference was not significant. Both groups had similar rates of wound infections and pneumonia. Similar results were seen in 117 post-pancreatic resection patients in New York [63].

Reducing the amount of glucose and protein in the TPN admixture did not affect the infection rate in 40 patients with a variety of underlying conditions. Six of 21 hypo-caloric TPN patients developed infections as did 10 of 19 standard TPN patients. This difference is not statistically significant due to the small sample size [64]. Thus, there is no data to support the idea that increased delivery of nutrients intravenously alone increases the rate of non-catheter infection, but the size of the samples and the duration of the TPN may be an important variable in these studies. Enteral feedings seem to protect against infection.

There are several potential explanations for this apparent protection provided by enteral feedings. There is evidence reviewed by Kudsk [65] that levels of IgA and numbers of circulating lymphocytes from gut-associated lymphoid tissue are strongly affected by enteral feeding. These lymphocytes migrate to non-gut tissues such as the lung and can alter the immune response in non-gut locales. The neuroendocrine system of the gut also affects the regulation of inflammation outside of the gut. This neuroendocrine system is modulated by the presence of dietary constituents [66,67]. The value of enteral feeding may be thus dependent on cellular and neuroendocrine factors.

### The value of nutritional supplements in the critically ill

There are many nutritional additives which are hypothesized to improve host defenses by boosting immunity and are called by some immuno-nutrients [68]. A meta-analysis compared published studies using two different "immunologically enhanced" enteral feedings with standard enteral feeding mixtures. These two "enhanced" mixtures had increased amounts of arginine, omega-3 fatty acids and nucleotides when compared with standard feedings. One of the feeding mixtures had added glutamine as well [69]. All studies analyzed enrolled critically ill medical and surgical patients. Although there was no detectable mortality benefit to enhanced feedings, there was an overall reduction in infectious risk to 0.60 (p < 0.005). No statistically significant infectious benefit could be found if only medical patients were analyzed (p = 0.30).

When studies enrolling only cancer patients are analyzed, no significant benefit of enhanced enteral nutrition was found in reducing nosocomial pneumonia. Effects on other infections were not analyzed. There was no significant effect of enhanced feeding on length of hospital stay or mortality [70]. If the enhanced feedings were started 7 days before cancer surgery and continued after the surgery, there was evidence of an infectious benefit in 206 Italian patients [71]. The pneumonia rate was more than twice as high in those receiving non-enhanced nutrition and the overall infection in the standard nutrition group was 30% compared to 14% in the enhanced nutrition group (p = 0.009).

When a routine hospital diet feeding was compared with a diet supplemented with 22.5 grams of extra protein and extra calories, there was no infectious risk benefit for stroke patients. A large multinational trial involving 4,023 patients showed no difference in pneumonia or urinary tract infection rates. The rate of developing pressure ulcers was not altered by supplementation either [72].

An innovative form of nutritional supplement was given to Hungarian patients with pancreatitis [73]. Twenty-three control and 22 experimental patients received nasojejunal feedings for 7–10 days. The control group also received 10 grams of oat fiber twice a day as well as 10 billion heat-killed *Lactobacillus plantarum*. The experimental group received the oat fiber and living bacteria. Seven control patients and only 1 experimental patient had a positive pancreatic aspiration culture (p = 0.05). Seven control patients required surgery to control sepsis compared to one experimental patient. The hypothesis that the composition of the enteral flora affects the rate of sepsis is supported by this study.

There is considerable literature regarding the amino acid glutamine's ability to preserve enterocyte integrity and reduce bacterial translocation from the gut [74]. When Dutch trauma patients received enteral nutrition or glutamine-supplemented enteral nutrition (which was iso-nitrogenous and iso-caloric), the glutamine supplemented group showed a definite infectious benefit [75]. There were fewer pneumonias (17% vs 43%) and bacteremias (9% vs 38%). A study of Spanish ICU patients with systemic inflammatory response syndrome yielded similar results for enteral glutamine supplementation [76]. The pneumonia incidence was about halved in the glutamine group (p = 0.04), although the incidence of bacteremia was unaffected by glutamine supplementation.

Glutamine need not apparently be given enterally to detect an infectious benefit. Burn patients receiving intravenous glutamine were compared with those receiving an iso-nitrogenous amino acid mixture. Only 1 of 12 glutamine patients had a Gram-negative bacteremia compared to 6 of 14 control patients (p = 0.04). The Gram-positive bacteremia and fungemia rates were unaffected by glutamine infusion [77]. Intravenous supplementation glutamine decreased bacteremias in bone marrow transplant patients when compared with parenteral nutrition without glutamine supplementation [78].

Table 2 summarizes the effect of various nutritional interventions on infections in hospitalized patients.

**Table 2:** Summary of randomized, controlled trials on the impact of nutritional interventions on infections in hospitalized patients

**Intervention**	**Effect**	**Reference**
Preoperative TPN	Increased infection incidence	40
Early vs late enteral nutrition	Decreased rate of infection in trauma and surgical patients	41,44,45,47
Early vs late enteral nutrition	No effect on infection incidence in stroke patients	48
Early vs late enteral nutrition	Increased infection rate in intubated patients in medical ICU	49
TPN vs enteral nutrition	Increased rate of infections	57–60
Immunologic feedings	No effect on infection incidence	70–72
Glutamine supplementation	Decreased infection incidence	75–78

## Conclusion

The literature concerning nutrition and infectious diseases in the developed world contains mixed support for ideas that nutritional supplements serve to prevent infectious diseases. While there is good evidence that enterally feeding those who have sustained surgical or accidental trauma prevents infections, other circumstances may implicate nutrition as a risk factor for infection rather than as a benefit. The following general points can be supported:

1. There is inconsistent evidence that healthy elderly and diabetic people can decrease their infectious risk by taking multivitamins and micronutrient supplementation.

2. Cranberry juice or concentrate can reduce bacteruria in women.

3. Yogurt or lactobacilli have an inconsistent effect in treating or preventing diarrhea.

4. Nutritional supplements offer little benefit in preventing infection in those already ill. The exception may be glutamine supplementation.

5. Enteral feeding is superior to parenteral feeding in lessening infectious risk.

6. Early enteral feeding in trauma and surgical patients prevents infections.

7. There is no best site for the delivery of enteral feedings. Gastric feedings do not appear to increase the risk of aspiration pneumonia when compared to small bowel feedings.
